# Keeping in Touch with Context: Non-verbal Behavior as a Manifestation of Communality and Dominance

**DOI:** 10.1007/s10919-018-0279-2

**Published:** 2018-03-26

**Authors:** Maciej Sekerdej, Claudia Simão, Sven Waldzus, Rodrigo Brito

**Affiliations:** 1 Institute of Psychology, Jagiellonian University, ul. Ingardena 6, 30-060 Kraków, Poland; 2 CUBE - Católica Lisbon School of Business and Economics, Universidade Católica Portuguesa, Lisbon, Portugal; 3 Instituto Universitário de Lisboa (ISCTE-IUL), Lisbon, Portugal; 4 Lusófona University, Lisbon, Portugal

**Keywords:** Touch, Communality, Dominance, Haptic behavior

## Abstract

This research investigated the influence of observed touch on the perceptions of communality and dominance in dyadic interactions. We manipulated four key situational features of haptic behavior in two experiments: the initiation, reciprocity, the degree of formality of touch (Studies 1 and 2), and the context of the interaction (Study 2). The results showed that the default perception of touch, irrespective of whether it is initiated or reciprocated, is the communal intention of the toucher. Furthermore, the initiation of touch was seen as an act of dominance, particularly, when the contact between the actors was primed as being hierarchical. Reciprocation neutralized the perceived asymmetry in dominance, but such inferences seemed to hinge on the fit of the touch with the context: reciprocation of formal touch reduced the asymmetry in the hierarchical context, whereas reciprocation of informal touch reduced the asymmetry in the non-hierarchical context.

## Introduction

Touch is a fundamental but complex behavior in daily social life. Touch may take a variety of forms and have a variety of meanings. It can be spontaneous, forced, overbearing, instrumental, ritualistic, or caring, to name just a few. Touches vary in duration and may involve a number of different body parts. People may touch each other simultaneously (e.g., hugs) or there may be a certain order of touching (e.g. someone reaches out his/her hand first, someone accepts it, or not, and so forth). We report two studies in this paper that tested how different qualities of touch convey essential messages in human relationships, in particular, communality and dominance, depending on the context of the interaction.

Haptic behavior (i.e., touching) conveys proximity and intimacy that help to establish and maintain a close relationship (Chopik et al. 2014; Simão and Seibt 2015; for review see Jakubiak and Feeney 2016). Neuroendocrinological research shows that touch releases oxytocin and endorphins, which are biological correlates of social connection (Dunbar 2010; Uvnäs-Moberg et al. 2015); it also facilitates the release of dopamine, which underlies our experience of sensory pleasure (cf. e.g., Keltner 2009). Other findings suggest that touching generally enhances prosocial behavior; it influences helping behavior (Cialdini et al. 1997) and increases compliance (Guéguen 2002; Willis and Hamm 1980).

On the other hand, some forms of touch can also communicate, deliberately or unintentionally, hierarchy-related messages that can influence observers as well as the actors themselves. For example, there is evidence showing that the type of touch—formal, informal, intimate—moderates the relationship of touch with power and status (Hall et al. 2005). Furthermore, there is a body of research showing that the observed initiation of touch frequently leads to a difference in the perceived power/dominance of dyadic interactions (cf. e.g., Eibl-Eibesfeldt 1989; Goffman 1967; Goldberg and Katz 1990; Hall et al. 2005; Henley 1973, 1977; Hertenstein et al. 2006; Major 1981; Major and Heslin 1982; Pisano et al. 1986; Summerhayes and Suchner 1978). While there is some agreement about the effect of the initiation of bodily contact, it is still not clear whether reciprocal touch can restore a disturbed power relationship between the two persons involved. Some authors argue that reciprocity neutralizes the perceived asymmetry (e.g., Henley 1973), but limited experimental evidence seems to dispute this (Goldberg and Katz 1990).

We intended to integrate these findings in the current research and to determine the boundary conditions for the effects of perceived intimacy and power that touch elicits in observers by combining four different situational features: initiation, reciprocity, formality of touch, and the context of the interaction. The present research is based on the idea that relational meaning is not just a product of touch, but instead, is a product of a combination of the features of touch. More specifically, we draw on Relational Models Theory (RMT; Fiske 1991, 1992) and conformation theory (Fiske 2000, 2004), according to which touch is part of the conformation system of communal relations, because it suggests a connection between bodies that creates an experience of unity and communality. On the other hand, when one person initiates the touch, and another receives the touch, this can be construed as a sign of asymmetry, which is characteristic of hierarchical relations, and thereby, can create the impression of dominance. We therefore suggest that both communality and dominance can be conveyed by the expansion of the self into the other, i.e., via touch. We present two studies testing specific hypotheses on how touch can affect perceptions of communality and dominance in dyadic interactions. In order to test this, we exposed participants to a video clip in which a male actor touched another male actor formally or informally and the touched actor either reciprocated the touch or did not. Then, we measured how the relationship between the persons who had engaged in the touching activity was perceived.

## Study 1

We hypothesized that the initiator of touch is perceived to express both (*H1*) a communal intention in the relationship with the other through physical bonding (e.g., Fiske 2004), particularly when the touch is informal, and (*H2*) dominance within that relationship (cf. *touch privilege*, Henley 1973). Reciprocity, on the other hand, should potentially re-establish an equilibrium and undo the asymmetry of dominance. We, therefore, expected that reciprocating touching behavior would (*H3*) increase the perceived dominance of the reciprocator, due to regaining the initiative, and (*H4*) increase the perceived communality of the reciprocator, appearing as a gesture of acceptance, particularly when the touch is informal.

### Method

#### Participants

We recruited 172 participants (119 females) with a mean age of 25.04 (*SD* = 7.97; range 17–69 years) for an online study using the mailing list of a Portuguese university. We aimed to obtain at least 140 participants, based on an a priori power analysis with a small effect size (*f *= 0.15) and power = .80 (Faul et al. 2007).

#### Procedure, Design, and Manipulation

When participants followed the link to the study, they read instructions saying that the study was about the formation of impressions of social interactions, and that they would see a short video clip (ca. 1 min) showing an interaction between two men, after which they would complete a questionnaire concerning the clip they had just seen. Different clips were used to represent the manipulation of type of touch and the reciprocity of the touching in a 2 (type of touch: informal vs. formal) × 2 (reciprocity of touch: yes vs. no) + 1 (no touch) between-subjects design. Our definition of formality of a touch was based on implied intimacy of touch between male interactors. A pretest conducted with the students of the same university showed that the least intimate touch among male colleagues, apart from a handshake, is a touch on the shoulder, and that the most relatively intimate touch, if the interacting individuals are sitting, is a touch on the knee.

The clips were filmed with two professional male actors who sat diagonally to each other at the same distance from the camera, with a simple table and a blank wall directly behind them. The actors conducted a conversation that they rehearsed from a prepared script, but this conversation was muted and unintelligible. The clips were edited by putting together the same segments of the non-manipulated part of the clips, and different segments of the manipulated parts. In the touch condition, one of the actors touched the other one midway through the conversation for one second. In the informal touch condition, the actor put the palm of his hand on the knee of the other actor. In the formal touch condition, the actor put his hand on the shoulder of the other actor. In the touch reciprocation condition, the second actor reciprocated this touch with the same level of formality a few seconds later. The order of the touches the actors gave each other and their sitting positions (left–right) were counterbalanced between the clips to control for actor and position effects what makes a pool of four videos per touch condition altogether. In the no-touch condition, we counterbalanced only for the sides the actors were seated, making two videos altogether. However, each participant saw only one random clip, according to the condition s/he was assigned to (sample clips can be found at https://osf.io/r5e4j). The clips were followed directly by the questionnaires; to see and fill in the questionnaires the participants had to watch the clip to the end. Finally, the participants were thanked and debriefed via email.

#### Measures

Participants rated both the initiator and the receiver of the first touch (or targets acting in the corresponding roles in the no touch conditions) on 7-point scales, ranging from 1 (*totally disagree*) to 7 (*totally agree*). The measures were based on Relational Models Theory (Fiske 1991, 1992), particularly on the models of Communal Sharing (communality) and Authority Ranking (dominance). They rated communality (whether the target person was warm toward, cold toward [reversed coded], close to, had affection for, was insensitive to [reversed coded], was supportive of, cared for, and liked the other person; αs > .84), and dominance (whether the target exercised power over, was superior to, dominant to, supervised, consulted, or protected the other person, and whether the other person was protected by and submissive to the target; αs > .83). Hence, each actor was evaluated twice—in terms of communality and dominance—by each participant. The order of the measures were counterbalanced.

### Results

The descriptive results are shown in Table 1.

**Table 1:** Ratings of communality and dominance in the different experimental touch conditions in Study 1

Target	Condition	95% confidence interval for mean			
		Mean	Lower bound	Upper bound	*SD*
*Communality ratings*					
Initiator	No touch	4.18	3.92	4.44	0.70
	Formal	4.57	4.20	4.93	1.10
	Informal	4.98	4.54	5.43	1.23
	Formal reciprocated	4.55	4.05	5.04	1.22
	Informal reciprocated	5.11	4.83	5.40	0.97
Receiver	No touch	4.70	4.42	4.98	0.74
	Formal	4.63	4.30	4.97	1.03
	Informal	4.62	4.24	4.99	1.04
	Formal reciprocated	4.53	4.20	4.86	0.81
	Informal reciprocated	4.96	4.67	5.25	0.97
*Dominance ratings*					
Initiator	No touch	3.31	2.94	3.69	1.01
	Formal	3.02	2.64	3.40	1.15
	Informal	3.41	3.02	3.79	1.07
	Formal reciprocated	3.44	2.91	3.98	1.32
	Informal reciprocated	3.27	2.99	3.56	0.95
Receiver	No touch	3.59	3.20	3.97	1.03
	Formal	3.33	2.97	3.68	1.08
	Informal	3.73	3.38	4.09	1.00
	Formal reciprocated	3.42	2.96	3.89	1.14
	Informal reciprocated	3.41	3.16	3.67	0.86

#### Initiation, Reciprocation, and Intimacy of Touch as a Communal Signal

**Fig. 1:**
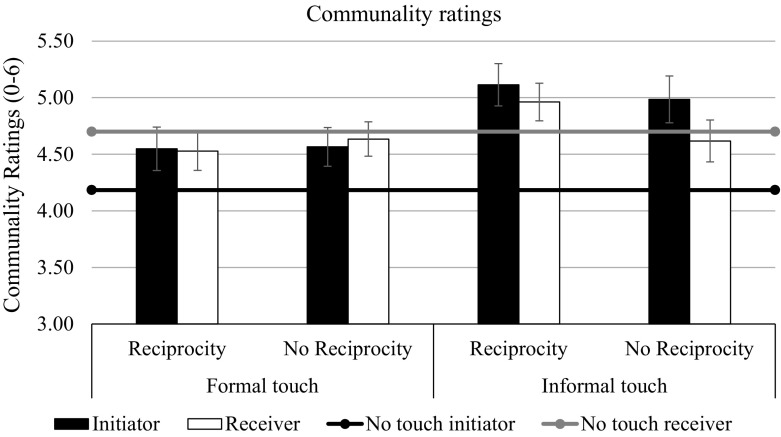
Communal ratings as a function of touch and reciprocity in Study 1. Bars represent standard errors

We initially conducted a repeated-measures analysis of variance (ANOVA) on the communality ratings, with initiation of touch as a within-subject factor (target: touch initiating vs. receiving person) and touch condition as a between-subject factor (touch condition: informal reciprocated touch vs. formal reciprocated touch vs. informal non-reciprocated touch vs. formal non-reciprocated touch vs. no touch). We found a significant main effect of touch condition on perceived communality, *F*(4, 167) = 3.54, *p* = .008, η_p_^2^ = .08, and a marginally significant interaction between target and touch condition, *F*(4, 167) = 2.16, *p* = .076, η_p_^2^ = .05 (see Fig. 1).

We performed planned contrasts to test our hypotheses using the LMATRIX and MMATRIX specifications in the GLM procedure of SPSS 22. The first contrast, which tested whether initiating touch increased the perceived communality of the initiator (touch condition coding 1 1 1 1 -4, target coding 1 0) was significant, *F*(1, 167) = 8.49, *p* = .004, η_p_^2^ = .05. The second contrast, which tested whether informal touch increased the perceived communality of the initiator compared to formal touch (touch condition coding 1 -1 1 -1 0, target coding 1 0), was also significant, *F*(1, 167) = 7.41, *p* = .007, η_p_^2^ = .04. An exploratory third contrast testing whether reciprocation of the touch affected the perceived communality of the original initiator (touch condition coding 1 1 -1 -1 0, target coding 1 0) was not significant, *F*(1, 167) = 0.10, *p* = .76, η_p_^2^ < .001. Thus, observing a touch, particularly an informal touch, indeed increased the perceived communality of the person who initiated the touch, irrespective of whether it was reciprocated or not. As can be seen by the means in Table 1 and Fig. 1, the pattern is slightly different for the ratings of the receiver of the first touch. Reciprocating a formal touch (i.e., touching back formally after previously having been touched) did not increase perceived communality; only reciprocating informal touch did. Accordingly, the contrast testing the informal reciprocating touch condition against all other conditions (touch condition coding 4 -1 -1 -1 -1, target coding 0 1) was significant for the reciprocator, *F*(1, 167) = 4.42, *p* = .037, η_p_^2^ = .03.

#### Initiation, Reciprocation, and Intimacy of Touch as a Dominance Signal

The same repeated-measures ANOVA on the dominance ratings did not find any significant effects, for the omnibus tests of the main effects, the interaction, or any of the planned contrasts testing our hypotheses.

### Discussion

Touching, in particular informal touching, generally made participants perceive the actors as being more communal. Specifically, the initiation of the touch decisively increased the perceived communality of the initiator, regardless of the type of the touch. However, the informal touch (knee) was a significantly better predictor of that perception, although this effect was somehow weaker. Reciprocity affected the perception of the (initial) receiver, but not the initiator, in the informal condition only: when the receiver reciprocated an informal touch, he was perceived as more communal than when he did not. The perceived communality of the initiator was higher in the informal touch condition and subsequent touches did not change that initial perception. The receiver, on the other hand, modified his image by returning an informal touch, which seemed to be more meaningful than a formal touch, and may be seen as a positive response or even as retaking the initiative.

Neither the type of touch nor reciprocity had an effect on the perception of the dominance of the initiator or the receiver. The latter finding requires cautious interpretation. Why were there no changes in the perceived dominance of either the initiator or the receiver, which is what would be predicted given the findings of previous studies? We reason that when observers do not have any clues regarding the observed interaction, they might hesitate to make relational inferences in terms of asymmetries (particularly dominance asymmetries), and therefore, if the observed gesture is not openly aggressive or intense (cf. e.g., Hertenstein et al. 2006), they tend to perceive it, by default, as a sign of communality. For instance, whereas RMT (Fiske 1991, 1992) assumes that touch is clearly part of the default mode of constituting communal sharing relations (i.e., not requiring contextualization), it is plausible that additional layers of relational meaning might require summoning additional contextual information. Indirect evidence for such contextual inferences comes from research testing the effect of actual touch on communal behavior, which has been found to be positive in a non-competitive context, but negative in a competitive context (Camps et al. 2013). Also, Heslin and Boss (1980) found that in a context devoid of any clear hierarchy-related cues (airport encounters), most touching behavior is perceived as a manifestation of closeness and intimacy. Participants in the current study might have lacked such additional contextual cues, so they did not perceive touching as dominance. Another explanation is that because they were students, they might have seen the interaction between the actors as being between students, and in a student environment hierarchical relationships are rare, or at least not the norm. Thus, we suppose that, overall, our participants saw the actors principally from a communal perspective, or at least not a hierarchical perspective, so the initiation of the touch was not perceived by them as a sign of dominance.

This interpretation strongly emphasizes context. Hence, in a more hierarchical context, the perception of the dominance of the initiator and receiver might have been different. In a hierarchical (e.g., organizational) context, the interaction between the actors presumably would not have been seen as, say, ‘two students discussing a paper’, but more as an official meeting, where one person is a superior and the other is a subordinate. Consequently, the initiation and reciprocation of a touch would have had different meanings, specifically in terms of indicating the presence or absence of dominance in the relationship. We decided to test this hypothesis by manipulating the context of the interaction in Study 2.

## Study 2

The results of previous research suggest that, in well-specified social contexts, certain types of touch can play different roles in expressing power and status. For instance, Goldstein and Jeffords (1981) found that in formal contexts (i.e., when the hierarchical status of the interactors is clearly defined), formal touches, as opposed to informal touches, were more frequently initiated by persons of lower status. Likewise, a study by Hall (1996), which manipulated hierarchical context, found that higher-status persons were more likely to be the initiators of different kinds of touch (formal and informal), presumably to display status, whereas lower-status persons were more likely to initiate formal than informal touches, presumably to gain status.

While the results of previous research at least partially account for the way that actual touching is adjusted to fit the relational context in which it takes place and one’s position within that relational context, a more fine-grained analysis is necessary when examining the inferences that are drawn from observed touch. On one hand, observing only the initiation of touch might allow for relatively robust inferences about the relational intentions or motivations of the initiator. The results from Study 1, however, seem to indicate that such robust inferences are made only in terms of the expression of communal intentions (*H1*), but not in terms of dominance intentions (*H2*). The latter might demand additional contextual information in order for an inference to be made.

Yet, observing whether or not the initial touch is reciprocated might not only trigger inferences about the reciprocator’s communal or dominance intentions (*H3* and *H4*), but also allow additional relational inferences to be made, for instance, about the presence or absence of asymmetries in the relationship. Given that such additional layers of relational meaning should be highly context-dependent, we propose that reciprocating a touch should be more meaningful for observers when the type of touch (formal vs. informal; touching back—i.e., reciprocal touching) fits the context of the interaction (e.g., hierarchical vs. non-hierarchical). Thus, we also expected that (*H5*) in a hierarchical context, the reciprocation of a formal touch, as ‘both polite and inherently equalizing’ (Hall 1996, p. 41), leads to the neutralization of the relational asymmetry between the original initiator and receiver, more than does the reciprocation of an informal touch. In contrast, in a non-hierarchical context, (*H6*) reciprocating an informal touch, which is more intimate in nature than a formal touch, is more meaningful, and therefore, reduces asymmetries more than does reciprocating a formal touch.

### Participants

We recruited 190 participants (127 females; mean age = 27.4, *SD* = 9.2) for an online study using the mailing list of a Portuguese university. The data of 27 subjects from the initial sample (*N *= 217) were excluded from the analyses because they reported a nationality other than Portuguese,^1^ they did not indicate any nationality, or they had participated in Study 1. We aimed to obtain at least 190 participants based on an a priori power analysis with a small effect size (*f *= 0.15) and power = .80 (Faul et al. 2007).

#### Measures

We employed the same questionnaire used in Study 1 to measure communality and dominance (αs > .82).

#### Procedure, Design, and Manipulation

The procedure of this study followed the procedure used in Study 1, with one exception: we introduced a manipulation of context: hierarchy versus communality. Having opened the link of the study on their PCs, participants in the *hierarchy* or [non-hierarchy/*friendship*] priming conditions read the following instructions:

Dear Participant, thank you for taking part in this short survey. We want to know how different individuals perceive *a ‘person in authority’* [*friendship*]. At the beginning of this questionnaire, we will ask you to give a short description about what you think *a ‘person in authority’* [*friendship*] is all about. Research suggests that with only little information, we can create concrete assumptions and evaluations about people. Therefore, we ask you to answer all the questions, even if you think that you do not have enough information. Please write down a few sentences describing your image of *a ‘person in authority’* [*friendship*].

Then, participants watched the same video clips used in Study 1, followed directly by the questionnaire containing the measures. Finally participants were thanked and debriefed via email.

### Results

#### Initiation, Reciprocation, and Intimacy of Touch as a Communal Signal

The descriptive statistics for the communality ratings can be seen in Table 2.

**Table 2:** Ratings of communality in the different experimental contexts and touch conditions in Study 2

Target	Condition	95% confidence interval for mean			
		Mean	Lower bound	Upper bound	*SD*
*Non*-*hierarchical context*					
Initiator	No touch	3.99	3.50	4.47	0.98
	Formal	4.77	4.35	5.18	0.99
	Informal	4.89	4.36	5.43	0.99
	Formal reciprocated	4.80	4.31	5.29	0.95
	Informal reciprocated	4.51	4.05	4.96	1.07
Receiver	No touch	4.24	3.75	4.74	0.99
	Formal	4.11	3.86	4.38	0.62
	Informal	4.26	3.71	4.82	1.05
	Formal reciprocated	4.34	3.95	4.73	0.75
	Informal reciprocated	4.54	4.06	5.02	1.13
*Hierarchical context*					
Initiator	No touch	3.09	2.88	3.29	0.39
	Formal	5.14	4.73	5.55	0.88
	Informal	5.20	4.86	5.53	0.77
	Formal reciprocated	4.50	4.06	4.94	0.80
	Informal reciprocated	5.06	4.73	5.39	0.64
Receiver	No touch	3.81	3.39	4.24	0.79
	Formal	4.16	3.68	4.64	1.03
	Informal	4.39	4.08	4.71	0.73
	Formal reciprocated	4.53	4.03	5.03	0.90
	Informal reciprocated	4.57	4.09	5.06	0.94

**Fig. 2:**
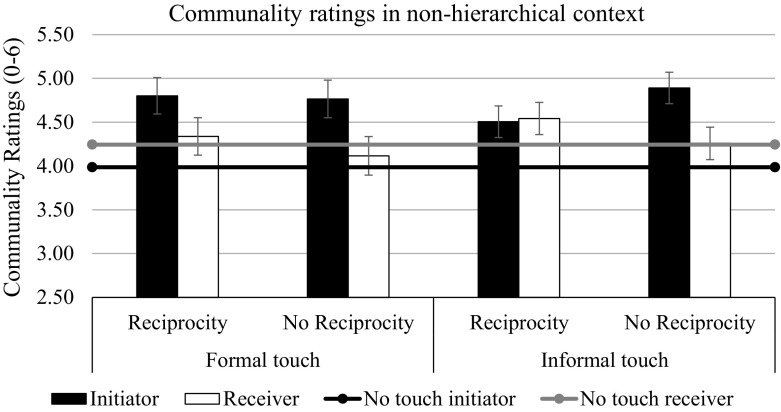
Communality ratings as a function of touch and reciprocity in non-hierarchical context in Study 2. Bars represent standard errors

**Fig. 3:**
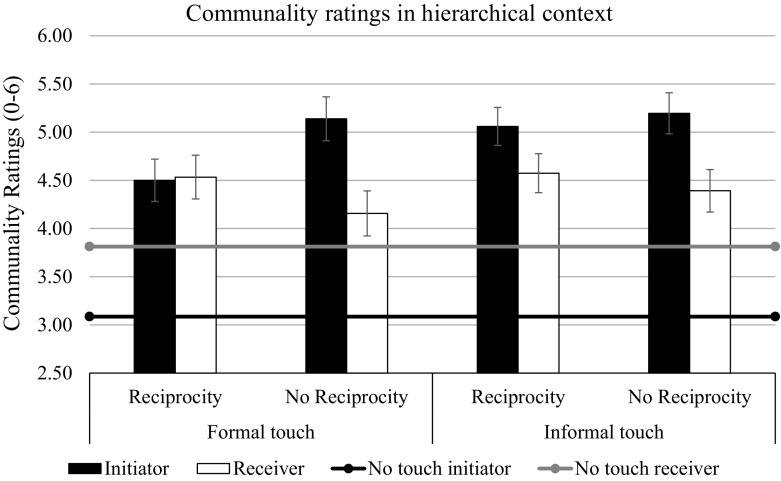
Communality ratings as a function of touch and reciprocity in hierarchical context in Study 2. Bars represent standard errors

We initially conducted a repeated-measures ANOVA with the initiation of touch as the within-subjects factor (initiating vs. receiving a touch) and touch condition (informal reciprocal touch vs. formal reciprocal touch vs. informal non-reciprocal touch vs. formal non-reciprocal touch vs. no touch) and context (non-hierarchy vs. hierarchy) as between-subject factors, with communality ratings as the dependent variable. The results revealed statistically significant main effects of touch condition, *F*(4, 180) = 9.29, *p* < .001, η_p_^2^ = .17, and target, *F*(1, 180) = 15.15, *p* < .001, η_p_^2^ = .08, as well as significant two-way interactions between target and touch condition, *F*(4, 180) = 9.28, *p* < .001, η_p_^2^ = .17 and context and touch condition, *F*(4, 180) = 2.60, *p* = .038, η_p_^2^ = .05 (see Figs. 2, 3). No other effect was significant.

We tested our hypotheses with planned contrasts using the LMATRIX and MMATRIX specifications in the GLM procedure of SPSS 22. The first contrast, which tested whether the initiation of touching increased the perceived communality of the initiator (touch condition coding 1 1 1 1 -4, target coding 1 0) was significant, *F*(1, 180) = 64.10, *p* < .001, η_p_^2^ = .25, replicating the findings from Study 1 and supporting H1. Exploratory contrast analysis showed that this effect interacted with context, *F*(1, 180) = 11.41, *p* < .001, η_p_^2^ = .06. It was stronger in the hierarchy priming condition, *F*(1, 180) = 60.12, *p* < .001, η_p_^2^ = .25, than in the non-hierarchy priming condition, *F*(1, 180) = 10.72, *p* = .001, η_p_^2^ = .06, because the person in the no-touch condition who was in the role usually taken by the initiator was seen as more communal after the non-hierarchy prime than after the hierarchy prime, *F*(1, 180) = 8.83, *p* = .003, η_p_^2^ = .05 (Table 2). Contrary to what we found in Study 1, the communality of the initiator was not significantly higher in the informal touch condition than in the formal touch condition (touch condition rating 1 -1 1 -1 0, target coding 1 0), *F*(1, 180) = 0.60, *p* = .44, η_p_^2^ = .003, not even in the condition with the hierarchy prime, *F*(1, 180) = 2.24, *p* = .14, η_p_^2^ = .01. However, the pattern of the means in this context was similar to that of the means in Study 1 (Table 2). Thus, in the current study, initiating touch increased the perceived communality of the initiator, independent of whether the touch was formal or informal. We then tested H4, whether reciprocating touch (condition coding 1.5 1.5 -1 -1 -1) would increase the perceived communality of the reciprocator (i.e., the receiver of the first touch; target coding 0 1). This contrast was significant, *F*(1, 180) = 5.94, *p* = .016, η_p_^2^ = .03, but it did not interact with context, *F*(1, 180) = 0.53, *p* = .47, η_p_^2^ = .003. There was also no significant difference between the reciprocation of the informal and the formal touch, *F*(1, 180) = 0.32, *p* = .57, η_p_^2^ = .002. Thus, contrary to what was found in Study 1, reciprocating both formal and informal touch increased the perceived communality of the reciprocator.

Finally, we tested the hypothesis that observers tend to infer equal levels of communality if the target used a type of reciprocal touch that fit well with the context (i.e., formal touch in the hierarchical context and informal touch in the non-hierarchy context), but not if the target used a type of touch that fit poorly with the context (i.e., the reverse). More precisely, we tested whether reciprocation would interact with the formality of touch and context when predicting differences in communality between the initiator and the receiver (target coding 1 -1). This contrast was only marginally significant, *F*(1, 180) = 3.05, *p* = .08, η_p_^2^ = .02. As predicted, however, touch reciprocation in the non-hierarchy context reduced the difference between the initiator’s communality and the reciprocator’s communality when touch was informal, *F*(1, 180) = 4.01, *p* = .047, η_p_^2^ = .02, but not when it was formal, *F*(1, 180) = 0.32, *p* = .57, η_p_^2^ = .002 (supporting H6). It had the same effect in the hierarchical context when touch was formal, *F*(1, 180) = 8.24, *p* = .005, η_p_^2^ = .04, but not when it was informal, *F*(1, 180) = 0.93, *p* = .34, η_p_^2^ = .005 (supporting H5; see Table 2, Figs. 2, 3).

To summarize, as predicted, touching increased the perceived communality of the touching person and this effect was relatively robust; that is, it was independent of the context triggered by priming and by type of touch (formal vs. informal). However, relationally meaningful inferences regarding reciprocal levels of communality as a result of reciprocal touching seemed to depend on whether the type of touch was seen as being appropriate for the context.

#### Initiation, Reciprocation, and Intimacy of Touch as a Dominance Signal

The descriptive statistics for the dominance ratings can be seen in Table 3.

**Table 3:** Ratings of dominance in the different experimental contexts and touch conditions in Study 2

Target	Condition	95% confidence interval for mean			
		Mean	Lower bound	Upper bound	*SD*
*Non*-*hierarchical context*					
Initiator	No touch	3.28	2.91	3.66	0.97
	Formal	3.38	3.05	3.70	0.89
	Informal	3.66	3.26	4.06	1.00
	Formal reciprocated	3.46	3.07	3.85	0.86
	Informal reciprocated	3.01	2.68	3.33	0.91
Receiver	No touch	3.52	2.88	4.16	1.29
	Formal	2.81	2.44	3.18	0.88
	Informal	2.91	2.44	3.37	0.88
	Formal reciprocated	3.07	2.62	3.53	0.88
	Informal reciprocated	2.99	2.64	3.35	0.84
*Hierarchical context*					
Initiator	No touch	3.64	3.24	4.04	0.54
	Formal	3.74	3.39	4.10	0.82
	Informal	3.98	3.65	4.32	0.83
	Formal reciprocated	3.94	3.53	4.36	0.45
	Informal reciprocated	3.58	3.19	3.97	0.66
Receiver	No touch	3.34	2.65	4.04	1.30
	Formal	2.91	2.46	3.37	0.97
	Informal	3.30	2.86	3.75	1.03
	Formal reciprocated	3.79	3.41	4.18	0.70
	Informal reciprocated	3.29	2.67	3.90	1.20

**Fig. 4:**
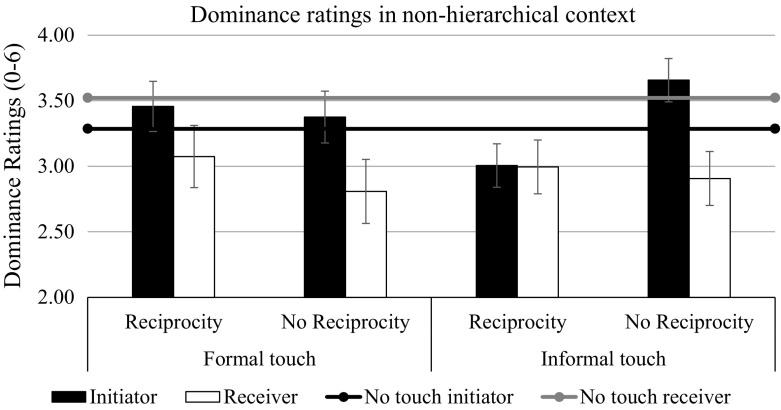
Dominance ratings as a function of touch and reciprocity in non-hierarchical context in Study 2. Bars represent standard errors

**Fig. 5:**
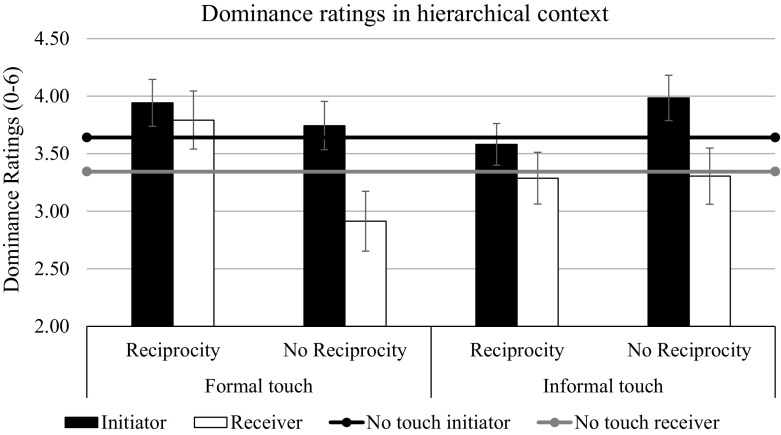
Dominance ratings as a function of touch and reciprocity in hierarchical context in Study 2. Bars represent standard errors

The same repeated-measures ANOVA on dominance ratings (the dependent variable) found main effects of context, *F*(1, 180) = 10.07, *p* = .002, η_p_^2^ = .05, and target, *F*(1, 180) = 21.99, *p* < .001, η_p_^2^ = .11, and a two-way interaction between target and touch condition, *F*(4, 180) = 3.28, *p* = .013, η_p_^2^ = .07. Neither the main effect of touch condition, *F*(4, 180) = 1.74, *p* = .143, η_p_^2^ = .04, nor any other effects were significant, all *F*s < 1, all *p*s > .33 (see Figs. 4, 5). We performed planned contrasts again to test our hypotheses. Our major predictions were that touch would produce asymmetries in dominance between the initiator and the receiver, but these asymmetries would be eliminated by reciprocating the touch, particularly when the type of touch fit the context (*H5* and *H6*). Therefore, we performed hypothesis tests on the effects of the difference between the initiator and the receiver of the touch (target coding 1 -1), as well as exploring separate effects on the initiator’s dominance ratings (target coding 1 0) and the receiver’s dominance ratings (target coding 0 1). The first contrast, which tested whether differences in dominance (i.e., the initiator–receiver dominance difference) were larger in the non-reciprocal touch conditions than in all the other conditions (touch condition coding -1 -1 1.5 1.5 -1), was significant, *F*(1, 180) = 12.17, *p* < .001, η_p_^2^ = .06. Exploratory contrast analyses revealed that, specifically, non-reciprocated touch increased this difference by simultaneously increasing the perceived dominance of the initiator, *F*(1, 180) = 2.90, *p* = .090, η_p_^2^ = .02, and decreasing the perceived dominance of the receiver, *F*(1, 180) = 5.60, *p* = .019, η_p_^2^ = .03. As predicted, reciprocation eliminated this effect as the initiator–receiver dominance difference was much smaller in the reciprocated touch conditions than in the non-reciprocated touch conditions, *F*(1, 180) = 8.03, *p* = .005, η_p_^2^ = .05, which were not significantly different from the no-touch condition, *F*(1, 180) = 0.63, *p* = .43, η_p_^2^ = .003.

Our ‘fit’ hypothesis found only partial support, as the interaction between the formality of the touch, reciprocation, and context had no significant effect on the initiator–receiver difference *F*(1, 180) = 1.46 *p* = .23, η_p_^2^ = .010. In line with this hypothesis, however, touch reciprocation in the non-hierarchy context reduced significantly the difference between initiator dominance and receiver dominance when touch was informal, *F*(1, 180) = 4.51, *p* = .035, η_p_^2^ = .02, but not when it was formal, *F*(1, 180) = 0.29, *p* = .59, η_p_^2^ = .002 (supporting *H6*). In contrast, touch reciprocation reduced the difference between initiator dominance and receiver dominance in the hierarchical context only when touch was formal, *F*(1, 180) = 3.41, *p* = .066, η_p_^2^ = .02, not when it was informal, *F*(1, 180) = 1.25, *p* = .27, η_p_^2^ = .007 (supporting *H5*, see also Table 3). Interestingly, explorative contrast analyses revealed that the elimination of dominance asymmetry was due to decreased initiator dominance by informal touch reciprocation in the non-hierarchy context, *F*(1, 180) = 6.17, *p* = .014, η_p_^2^ = .03, and due to increased receiver (i.e., reciprocator) dominance by formal touch reciprocation in the hierarchical context, *F*(1, 180) = 6.54, *p* = .011, η_p_^2^ = .04.

## Discussion and Conclusion

This research investigated the influence of contextual haptic behavior on the conveyance of perceptions of communality and dominance in dyadic interactions. Our approach provides novel, integrative insights into the effects of perceived touch, showing that its relational meaning depends on the combination of four key situational features: initiation, reciprocity, the degree of formality of touch, and the context of the interaction. The findings show, first, that the default inference (i.e., independent of context) when observing touch is the communal intention of the touching person, be it the initiator or the reciprocator. Whether this inference depends on the type of touch (formal vs. informal) is not clear, as an interaction with type of touch was found in Study 1, but not in Study 2. Second, additional relational inferences about the presence or absence of asymmetries in dominance are possible from the observation of the full interaction (i.e., whether an initial touch is reciprocated or not). Unlike the default inference of communal intentions, however, these additional relational inferences seem to hinge on the fit of the touch with the context. Although the statistical interaction with context was not significant, the conditional effects clearly indicate that reciprocation of a formal touch is more relationally meaningful in a hierarchical context, equalizing the perceived hierarchy between the interactors, whereas the same is true for informal touch in the non-hierarchical context.

Specifically, the initiator is always seen as being more communal towards the receiver, which is in keeping with the limited laboratory findings that are available (Major and Heslin 1982; Pisano et al. 1986). When the context of the interaction is neutral, the initiator can enhance this perceived communality by informal touch (Study 1). However, when the contact is already primed as being close, the difference between formal and informal touch does not change the perception of the initiator’s communality (Study 2). It is quite plausible that observers of a formal touch in a non-hierarchical, tangentially communal context, such as friendship, will imagine the same two people touching informally too. In turn, the receiver in a neutral context can enhance his perceived communality by reciprocating an informal, but not a formal touch (Study 1). In a primed friendship situation (such as Study 2), any reciprocation can increase the perceived communality of the reciprocator (cf. field observations by Heslin and Boss 1980); however, only informal touch that fit the context, reduced the difference in communality between the actors.

Furthermore, the initiation of touch is seen as an act that expresses dominance, especially when the contact between the actors is primed as being in a hierarchical context (Study 2). However, as in previous studies (Goldberg and Katz 1990; Major and Heslin 1982) even in the non-hierarchical context in Study 2, the initiator was seen as more dominating than the receiver. This suggests that when observers know that some hierarchy is embedded in the context, their vital question shifts from ‘I wonder if they like each other…’ to ‘who is who?’. In that case, the toucher, as the one who takes the initiative, increases his/her perceived dominance, while simultaneously decreasing the perceived dominance of the receiver. This is in line with previous findings that showed touch was perceived as a particularly meaningful message of power in contexts in which some asymmetry of power was assumed, whether this was inferred from gender, dress, or apparent age (Henley 1973, 1977; Summerhayes and Suchner 1978) or objectively measured by social-professional indicators (Goldstein and Jeffords 1981).

The receiver, however, can neutralize that asymmetry by reciprocating the touch, which may be seen by observers as a sign of regaining the initiative, or at least of not being merely a passive participant in the interaction. Interestingly enough, we found that the non-default, relational meaning of a touch depends on whether it fits with the context. Although any reciprocation—as an assumed sign of agency—increased the perceived dominance of the reciprocator in Study 2, it was only the reciprocation of formal touch in the hierarchical context that reduced the asymmetry in perceived dominance between the actors, confirming previous findings (Hall 1996). Moreover, only the reciprocation of informal touch did the same in the non-hierarchical context in Study 2. This may also explain Henley’s (1973, 1977) observations that reciprocity indicates a reassertion of power and solidarity, which was not confirmed by Goldberg and Katz (1990). Henley’s conclusions were drawn mainly on the basis of informal fieldwork situations and informal touches, whereas Goldberg and Katz conducted their studies in formalized laboratory settings and used informal touches, which in this context turned out to be ineffective.

In conclusion, our goal was to show that the relational meaning of touch hinges heavily on the combination of four characteristics: initiation, reciprocation, type of touch, and situational context. The present findings suggest that even though touch is, overall, a relational form of behavior, one can decode the meaning of each relational message only when combining these specific characteristics. Besides, we have to admit that a perfect control of some other cues (e.g., subtle facial movements) was not possible in our studies. We did our best, however, to minimize such other factors by employing professional actors who were strictly and carefully instructed to limit their facial movements, and other nonverbal behaviour. Likewise, the clips were then carefully counterbalanced—for the order of touching, actors and sides—hence many confounding factors, were substantially neutralized.

For this and other reasons, we are aware that one should be careful not to extrapolate any isolated result too generally, as these characteristics may vary across situations. In this vein, it is worth noting, for example, that our results may be gender-specific, as all our actors were male. Hence, further research on female and mixed-sex dyads would be required in order to verify this pattern of results. For instance, men touching women formally may be perceived as conveying a quite robust sign of dominance, as suggested by Henley (1977). In turn informal touch in mixed-sex dyads may be seen as much more sexually driven (i.e., it can influence the perception of communality) than in all-male dyads. As regards female dyads, one would expect that, e.g., informal touches, are less meaningful because there generally is greater social consent and tolerance among women of touching informally. However, cultural differences may play a crucial role here, which could constitute the subject of another whole line of research. Finally, as in most previous research on touch, we did not test the fifth presumably important factor, which is time. We suspect that the length of touch alters the relational meaning of haptic behavior regardless of the four factors we tested, which points to another important direction for future research.

## Footnotes

1 We did it particularly because of Erasmus students that participated in the study and whose Portuguese was potentially not good enough to understand the items.
